# A Research on the Macroscopic and Mesoscopic Parameters of Concrete Based on an Experimental Design Method

**DOI:** 10.3390/ma14071627

**Published:** 2021-03-26

**Authors:** Hui Su, Hongliang Li, Baowen Hu, Jiaqi Yang

**Affiliations:** 1 School of Water Conservancy and Hydroelectric Power, Hebei University of Engineering, Handan 056038, Hebei, China; 13082114687@163.com (H.S.); l18233324544@163.com (H.L.); demyang@163.com (J.Y.); 2 Hebei Key Laboratory of Intelligent Water Conservancy, Handan 056038, Hebei, China; 3 Beijing Key Laboratory of Urban Underground Space Engineering, Beijing 100083, China

**Keywords:** discrete element method, test design, parameter calibration, genetic algorithm, macro–mesoscopic parameters

## Abstract

Concrete is a composite material that has complex mechanical properties. The mechanical properties of each of its components are different at the mesoscopic scale. Studying the relationship between the macroscopic and mesoscopic parameters of concrete can help better understand its mechanical properties at these levels. When using the discrete element method to model the macro-mesoscopic parameters of concrete, their calibration is the first challenge. This paper proposes a numerical model of concrete using the particle discrete element software particle flow code (PFC). The mesoscopic parameters required by the model need to be set within a certain range for an orthogonal experimental design. We used the proposed model to perform numerical simulations as well as response surface design and analysis. This involved fitting a set of mapping relationships between the macro–micro parameters of concrete. An optimization model was established in the MATLAB environment. The program used to calibrate the mesoscopic parameters of concrete was written using the genetic algorithm, and its macro-micro parameters were inverted. The following three conclusions can be drawn from the orthogonal test: First, the tensile strength and shear strength of the parallel bond between the particles of mortar had a significant influence on the peak compressive strength of concrete, whereas the influence of the other parameters was not significant. Second, the elastic modulus of the parallel bonding between particles of mortar, their stiffness ratio and friction coefficient, and the elastic modulus and stiffness ratio of contact bonding in the interfacial transition zone had a significant influence on the elastic modulus, whereas the influence of the other parameters was not significant. Third, the elastic modulus, stiffness ratio, and friction coefficient of the particles of mortar as well as the ratio of the contact adhesive stiffness in their interfacial transition zone had a significant influence on Poisson’s ratio, whereas the influence of the other parameters was not significant. The fitting effect of the response surface design was good.

## 1. Introduction

No unified method is available to describe the macroscopic parameters of particles of geotechnical materials by using their microscopic parameters. Although laboratory experiments are a feasible approach, they have such problems as low efficiency, high cost, and a large dispersion in the results. Cundall established the discrete element method (DEM) to solve this problem [1]. The DEM is a numerical method to examine the mechanics of discontinuous media based on Newton’s second law of motion. It represents the given geotechnical material as a rigid particle model in which its mesoscopic and macroscopic parameters can be related. It is directly related to the geometrical characteristics of the assignment particles and particle contact between mesoscopic mechanics parameters of the mesoscopic parameters of macroscopic mechanical properties of the change means that significant changes.

The problem of matching the macro-meso parameters of materials is essentially one of mapping them. A few scholars have examined this relationship using the bonded particle model (BPM). Yang et al. [2] considered the relationship between the results of theory and a simulation from two perspectives to examine the relationship between the macro–micro parameters of the parallel-bond model (PBM). They found that the microparameters had significant influence on each macroparameter, and gave a simple quantitative relationship between them. Yoon [3] combined the Plackett–Burman design (PBD) and the central composite design (CCD) to study the relationship between the macroscopic and mesoscopic parameters of the contact bonded model (CBM) and gave the second-order response surface of each macroscopic parameter. Hanley [4], Nohut [5], and Chehreghani [6] used different experimental designs and methods of statistical analysis to study the relationship between the macroscopic and microscopic parameters of 2D and 3D models based on the PBM and gave different forms of quantitative Equation. The above research shows that numerical simulations, the design of experiment (DOE), and corresponding statistical analyses can be used to obtain the quantitative relationship between macroscopic and mesoscopic parameters. This significantly reduces the amount of work required for parameter matching, but considerable effort is still needed to find the best match. To solve this problem, Yoon [3] introduced an optimization method to automatically match macroscopic and mesoscopic parameters. Tawadrous et al. [7] matched the macroscopic and mesoscopic parameters of 3D models by using an artificial neural network algorithm. Wang et al. [8] combined the Python language and the particle flow code (PFC), and used the simulated annealing algorithm to match the microparameters of both the CBM and the PBM in the PFC.

Concrete is a composite material composed of aggregate, cement mortar and an interfacial transition zone. Under a load, the stiffness of each component varies greatly, resulting in uneven distributions of the stress and strain fields inside concrete. At the mesoscale, aggregate particles of concrete exhibit heterogeneity and contain a variety of microcracks. Examining the relationship between the macro - meso parameters of concrete is important for understanding its mechanical properties. Selecting appropriate microscopic parameters and reasonably controlling the scale of model calculations are key to obtaining ideal results in simulations using the particle discrete element model. No unified method is available for quantitatively determining the microscopic parameters of concrete for this model. Trial-and-error testing is typically used, in which the microscopic parameters are constantly adjusted until suitable results are obtained. However, this method is time consuming and labor intensive, and struggles to find the optimal matching scheme due to the randomness of the model. At present, the quantitative relationship between the macro–micro parameters of the DEM cannot be derived theoretically, and thus it cannot be directly used to calibrate them. This paper proposes a DEM based on the discrete element software PFC2D. The experimental design method is used to obtain a set of mapping relations between the macro–micro parameters of concrete, and a calibration program for the mesoscopic parameters is compiled to invert the macro–micro parameters.

## 2. Basic Parameter Setting and Model Building

### 2.1. Parameter Selection

**Figure 1:**
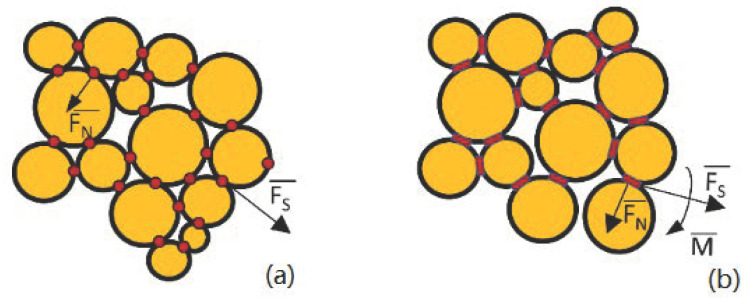
Contact model. (**a**) Linear contact bonding model (**b**) Parallel bonding model.

Concrete is an artificial mixed material with cement as the main cementing material. Sand, stone, and water are mixed in certain ratios, and the mixture gradually solidifies and hardens after molding injection, vibration, curing, and other procedures. It is a kind of non-uniform quasi-brittle material with complex mechanical properties. In this paper, we set the parameters of the model according to the difference in bond strength between aggregate and mortar. The particle contact model (Figure 1a) and particle bond model (Figure 1b) form the foundation of the PFC2D model. The stick model can only relay contact force, and the parallel bond model in 2D/3D represents the line/surface bonding rather than that at a point. This can better reflect the capability of concrete mortar to resist torque as well as the interfacial transition zone between particles. Therefore, in the concrete model in this paper, different contact models are set between aggregate and aggregate, and between mortar and mortar, so as to more truly reflect the actual performance of concrete. The parallel bonding model was used to simulate the mechanical behaviors of mortar and the aggregate, and the linear contact bonding model was used to simulate the contact-related behavior of coarse aggregate.

The strength and deformation characteristics of concrete were preliminarily characterized by using the three macroscopic parameters of peak strength, elastic modulus, and Poisson’s ratio, and were measured using uniaxial compression tests. The peak strength *σ_u_*, elastic modulus *E* and Poisson’s ratio *ν* were then used as test indices (objects), and the influence of the test factors (mesoscopic parameters of concrete) on them was examined. In the process of uniaxial compression in the particle discrete element model, *σ_u_* is taken as the stress corresponding to the apex of the stress-strain curve, *E* is the tangent slope of the curve when the stress reaches half the peak stress for the first time, and *ν* is the absolute value of the ratio of lateral strain to axial strain at this time.

In simulations of the uniaxial compression test, the geometric parameters (*L*,*W*,*n*, *R_max_*/*R_min_*), load parameters ($E_{c}^{w}$, $k_{n}^{w}/k_{s}^{w}$, $\nu^{w}$, $\mu^{w}$), parameters of the particles (ρ, *E_c_*, *k_n_*/*k_s_, μ*) and key binding parameters ($\bar{\lambda }$, ${\bar{E}}_{c}$, ${\bar{k}}_{n}/{\bar{k}}_{s},\;$ ${\bar{\sigma }}_{c},{\bar{\tau }}_{c}$) affect the results. Because the parameters of the particles (including particles of cement mortar (ball) and aggregate particles (clump)) and bonds are the focus of this study, the geometric and load parameters were controlled in advance. The compression of members in engineering is mostly prismatic, because of which prismatic specimens can better reflect the compressive capacity of concrete than cubic specimens. A model size of 200 × 100 mm^2^ was thus set. Porosity (*n*) was set to 0.12, the radius ratio of the particles of cement mortar (*R_max_*/*R_min_*) was 2.5, particle density (*ρ_ball_*) was 2000 kg/m^3^, and aggregate density was 2650 kg/m^3^. We preset the average radius of particles of cement mortar ( $\bar{R}$ ) to 0.5 mm. The bonding radius factor ( $\bar{\lambda }$ ) is normally set to one, and remains constant during the calculation; thus, it was not be considered. To reduce the number of unknown parameters, the stiffness parameters of the particles of cement mortar, their bonds, and the upper and lower loading plates were set to be consistent, that is, *k_n_*/*k_s_* = ${\bar{k}}_{n}/{\bar{k}}_{s}$ = $k_{n}^{w}/k_{s}^{w}$, *E_c_* = ${\bar{E}}_{c}$ = $E_{c}^{w}$. Parameters of the particles of aggregate were set to be consistent with those of contact stiffness, i.e., (*k_n_*/*k_s_*)*_g_* = (${\bar{k}}_{n}/{\bar{k}}_{s}{)}_{g}$ and (*E_C_*)*_g_* = (${\bar{E}}_{c}$)*_g_* given a wall loading rate ( $v^{w}$) of 0.02 m/s, the friction coefficient of the wall (*μ^w^*) was zero.

Finally, the three macroscopic parameters *σ_u_*, *E* and *ν* were determined as test indicators. Parameters of the particles of cement mortar and their bonds (*E_c_*, *k_n_*/*k_s_*, ${\bar{\sigma }}_{c},\;\;{\bar{\tau }}_{c}$, and *μ*), the aggregate ((*E_c_*)*_g_*, (*k_n_*/*k_s_*)*_g_*, *μ*_g_), and the interfacial transition zone and its bonds ((*E_C_*)*_j_*, (*k_n_*/*k_s_*)*_j_*, (${\bar{\sigma }}_{c}$)*_j_*, (${\bar{\tau }}_{c}$)*_j_*, *μ_j_*), a total of 13 mesoscopic parameters, were unknown. They were chosen as the experimental factors. *y* = *E*_c_, *k_n_*/*k_s_*, ${\bar{\sigma }}_{c},\;\;{\bar{\tau }}_{c}$, *μ*, (*E_c_*)*_g_*, (*k_n_*/*k_s_*)*_g_*, *μ*_g_, (*E_c_*)*_j_*, (*k_n_*/*k_s_*)*_j_*, (${\bar{\sigma }}_{c}$)*_j_*, (${\bar{\tau }}_{c}$)*_j_*, *μ_j_*.

The relationship is as follows:

(1) $$\sigma_{u}=f_{\sigma }\left(y\right)\;\;$$

(2) $$E=f_{E}(y)\;$$

(3) $$\nu =f_{\nu }(y)$$

As concrete is a complex three-phase composite material with obvious anisotropy, the microscopic parameters of the aggregate, cement mortar, and the weak interfacial transition zone all need to be considered. The interfacial transition zone features both the aggregate particles and the cement paste, where the distribution of the cement particles is affected by the aggregate surface at the parts in contact. Due to the presence of many original cracks in the interior, most of the damage under external load starts from the interfacial transition zone, because of which it is the weakest part of concrete. The strength of the zone affects the overall mechanical properties and failure characteristics of concrete [9] as well as the fluctuation in its stress–strain curve. Therefore, it must be considered to enable the model to represent empirical scenarios involving concrete. The aggregate is the “skeleton” of concrete that plays an important load-bearing role. In the PFC2D software, the thickness of the interfacial transition zone cannot be expressed because the bond between particles is set through contact. In this paper, the influence of the thickness of the interfacial transition zone on the mechanical properties of concrete was ignored.

The parameters of the model are shown in Table 1.

### 2.2. Particle Formation

The literature has shown that when the number of particles in the sample is greater than 2000, the peak axial stress of the given sample is relatively stable, and the precision of the parameters of concrete obtained using a simulation is acceptably close to that obtained through laboratory experiments [10], The particles in the sample were filled using the radius expansion method. The number *N* of particles to be placed was estimated according to Equation (4). The number of particles was calculated to be 22,000, which is acceptable:

(4) $$N=\left\lfloor \frac{\left(L\times W\right)\left(1-n\right)}{\pi R^{2}}\right\rfloor$$

where *L* is the height of the model (mm), *W* is its width (mm), *n* is its porosity (-), *R* is the average particle radius (mm), and   is the rounded down result of the calculation.

To restrict the magnitude of the overlap between the randomly generated particles, they were first produced at half the final particle size, and the porosity *n*_0_ and average radius *R*_0_ of the model were calculated. Then, the particle radius was amplified to obtain the desired particle size:

(5) $$\frac{V_{a}(1-n)}{\pi \bar{R^{2}}}=\frac{V_{a}(1-n_{0})}{\pi \bar{R_{0}^{2}}}$$

The amplification coefficient *m* is given by:

(6) $$m=\frac{\bar{R}}{\bar{R_{0}}}=\sqrt{\frac{1-n}{1-n_{0}}}$$

### 2.3. Aggregate Generation

Past work has used the CT scanning technique to simulate the aggregate, and the scanned images are subjected to threshold processing using third-party software to obtain the boundary information to establish the model. However, this often reflects only one or more conditions while ignoring the random aggregate model. This paper uses a random algorithm for generating a convex polygonal aggregate.

In this algorithm, the circle is used as the base to generate the aggregate to ensure that the model is convex polygonal. The number of points on the circle determines the number of sides of a convex polygon. To ensure the randomness of the number of edges of the generated aggregate, a minimum value and a maximum value were set for the number of points on the circle. The range of the number of edges of the convex polygon was determined by calculating the difference between them, which was then multiplied by a random number. To round up the results, the resulting integer was set as the number of points on the circle, which was also the number of sides of the convex polygon:

(7) $$N{=\;\mathrm{round}[N}_{\min}+range\times (N_{\max}-N_{\min})]$$

where *N* is the number of points on the boundary of the circle, *N*_min_ is the minimum number of points generated. *N*_max_ is the maximum number of points generated, range is a pseudo-random number uniformly distributed between zero and one, and *round* is a rounding of the entire function.

Assuming that one of the circles has radius *R* and coordinates (*X*,*Y*), the point coordinates (*X*_1_,*Y*_1_) on each circle are calculated by Equation (8):

(8) $$\left\{\begin{array}{c} X_{1}=\;X+Rcos\theta \\ Y_{1}=\;Y+Rsin\theta \end{array}\right.$$

where *θ* is the anticlockwise angle between the line of each point and the center of the circle and has a positive value along the *X*-axis.

Given the randomness to ensure the positional angle, the normal vector of the convex polygon contains information on internal damage that is used to determine the scope of the convex polygons generated after the outline. The aim is to build a set of algorithms to calculate the convex polygons of each edge point with respect to its internal normal vector.

Based on the proposed algorithm, multiple aggregate models were built. The fewer edges the aggregate model had, the sharper was its shape. The model was used to simulate an elongated aggregate. The more edges the aggregate model had, the smoother the shape of the aggregate was, and the more round it tended to be. This model was used to simulate the circular aggregate.

To simulate aggregate of any shape for the numerical analysis of concrete, multiple particles can be combined in the particle flow program to form a “block.” The block formed by a clump participates in the cyclic calculation as a rigid body. During the calculation process, the distance and contact force between the internal particles do not change with the steps of calculation, and the deformation of the block occurs only along the boundary. Previous studies have shown that the compressive strength of the aggregate is twice as high as that of cement mortar, and it is not damaged in the process of concrete compression. Therefore, the unbreakable unit “clump’’ is used as the object of generation of aggregate in the simulation. In the calculation cycle, contact between the particles inside the “block’’ is ignored, which reduces calculation time.

The range of sizes of the coarse aggregate used was 5~15 mm, and the Walraven Equation [11] (Equation (9)) was used to calculate its gradation. The percentage (*P_k_*) of the volume of coarse aggregate in the concrete specimen was 75%. The average gradation curve obtained by using a slice of the non-circular aggregate concrete specimen and subjecting it to image processing using the “equivalent particle size” (*D_equivalent particle size_* = 2(*S_arregate area_*/*π*)^0.5^) conformed to a 3D gradation curve and a 2D conversion curve. Therefore, the circular area of the aggregate was used as the equivalent area of the convex polygonal aggregate. Aggregate sizes of 5~7 mm, 7~9 mm, 9~11 mm, 11~13 mm, and 13~15 mm was used, and the results are shown in Table 2:

(9) $$\begin{array}{l} P_{C}(D<D_{0})=P_{K}[1.065{\left(\frac{D_{0}}{D_{max}}\right)}^{0.5}-0.053{\left(\frac{D_{0}}{D_{max}}\right)}^{4}-0.012{\left(\frac{D_{0}}{D_{max}}\right)}^{6}\\ -0.0045{\left(\frac{D_{0}}{D_{max}}\right)}^{8}+0.0025{\left(\frac{D_{0}}{D_{max}}\right)}^{10}] \end{array}$$

where *P_k_* is the percentage by volume of coarse aggregate in the concrete specimen, *P_C_* (*D* < *D*_0_) is the probability that the particle size *D* in the section is less than the size of the sieve *D*_0_, and *D*_max_ is the maximum aggregate particle size.

The number of particles within the range of each particle size is calculated according to the following equation:

(10) $$n_{i}=\left(p_{i}-p_{i-1}\right)A/A_{i}$$

where *n_i_* is the number of particles in a certain range, *p_i_* is the probability that the particle size in the section is smaller than the sieve in the Walraven Equation, and *A* and *A_i_* are the sectional area and aggregate area, respectively.

**Figure 2:**
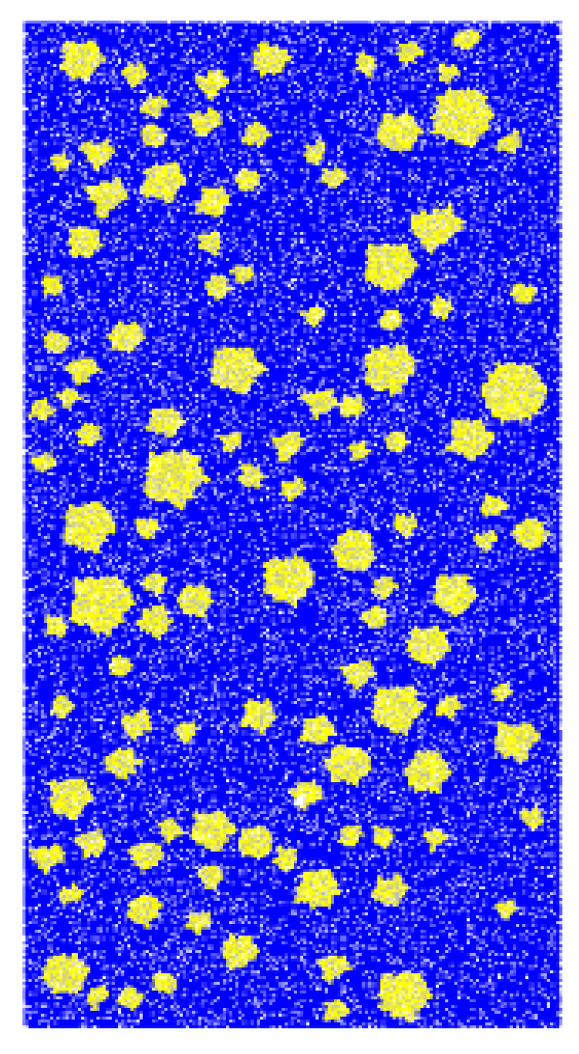
Discrete particle element model.

We set-up four to six polygons sides in MATLAB to generate a random aggregate model and imported it into the PFC2D model for use as a model of the concrete aggregate. The generation model is shown in Figure 2.

### 2.4. Verifying Model Feasibility 

Because the mesoscopic parameters cannot be directly obtained from experiments, numerical simulations were used, and the results were compared with those of experiments to verify the feasibility of the numerical model. According to the literature review, the elastic modulus of concrete increases with *E_c_*, and its peak strength increases with ${\bar{\sigma }}_{c},\;\;\mathrm{and}\;{\bar{\tau }}_{c}$. *E_c_* and *k_n_*/*k_s_* have a significant influence on the elastic modulus of concrete and its Poisson’s ratio. The compressive strength and elastic modulus of the interfacial transition zone should be lower than those of cement mortar by the same proportion [12,13]. Based on this, a trial-and-error method was used to model the mesoscopic parameters following a set of mesoscopic parameters (Table 3), with the macroscopic parameters of C30 concrete as reference (30 MPa, compressive strength; elastic modulus, 30 GPa; Poisson’s ratio, 0.2) to obtain the numerical macroscopic parameters *σ_u_* = 32.23 MPa, *E* = 28.37 GPa, and *ν* = 0.197. The error was very small, which shows that the numerical model is feasible.

## 3. Determining Research Methods and Research Interval

### 3.1. Research Methods

A preliminary analysis showed that to get a set of mapping relations between the mesoscopic parameters of concrete, 13 factors need to be considered on three test indices. Considering the nonlinear relationship between the macroscopic and microscopic parameters, the number of levels should be three or more. A long time and considerable effort are required to study the multi-level and multi-factor problems of a comprehensive test. The DOE is an efficient procedure for planning experiments so that the results obtained can be analyzed to yield valid and objective conclusions. It begins by determining the objectives of a given experiment and selecting the process variables for it. We used the DOE to arrange our test to reduce the time needed and applied it to determine the bonding between microparticles of the model during contact in a sensitivity analysis.

The test points selected by using an orthogonal test design are more representative and can give reliable research conclusions by significantly reducing the number of tests. This design can be used to screen out the microscopic parameters of concrete that have a significant influence on its macroscopic parameters, and to estimate the quantitative relationship between the two types of parameters. Although a screening test can achieve the same goal with fewer trials, it can select only two horizontal values for each factor and cannot consider nonlinear influences. Incorrect conclusions are thus easily drawn when the scope being considered is wide. We used the orthogonal experimental design for parameter sensitivity analysis.

**Figure 3:**
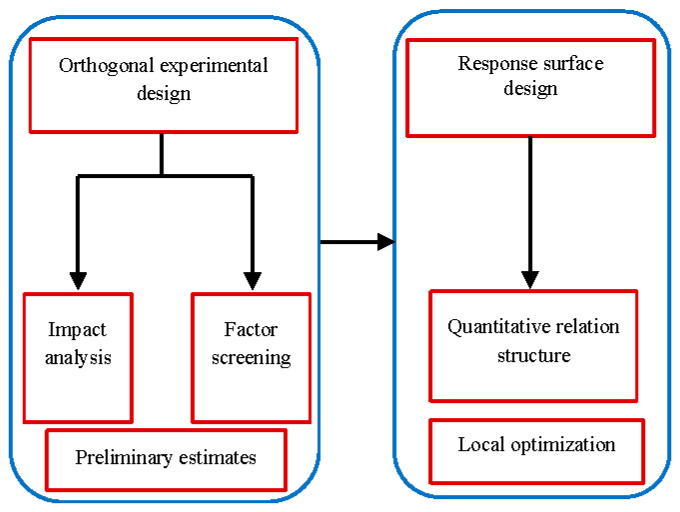
Idea of the experimental design.

Response surface design represents the nonlinear relationship between the test indices and factors. We used the second-order response surface equation. The experimental design idea is shown in Figure 3.

### 3.2. Determining the Research Interval

An orthogonal experiment was carried out to determine the first sites to arrange an orthogonal test table and generate the test scheme. The mesoscopic parameters of the C30 concrete used as reference, shown in in Table 3, were used.

## 4. Orthogonal Experimental Design

**Figure 4:**
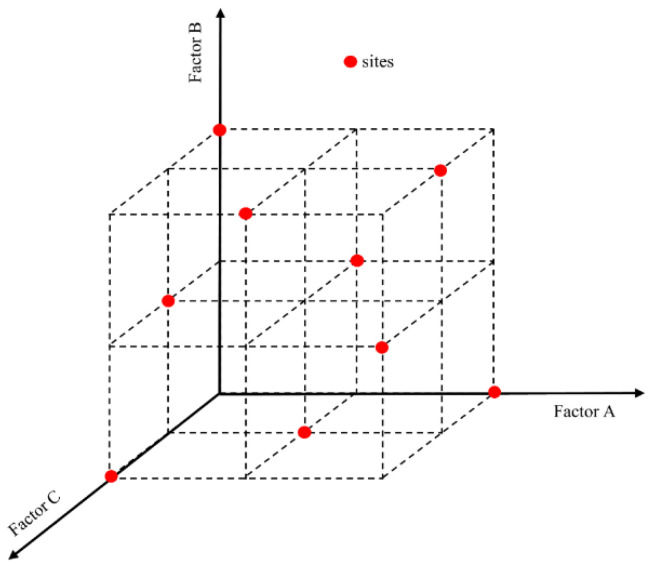
Distribution of test points in orthogonal experiments.

Orthogonal experiment design refers to the examination of multiple factors using the experimental design method. According to orthogonality, representative points are selected from the overall test that have the characteristics of uniform dispersion and neat comparison. Orthogonal experimental design is the main method of factorial design. When three or more factors are involved in the experiment that may interact, the test workload becomes large and difficult to implement. The orthogonal design of an experiment is a good choice in such cases. The main tool of orthogonal experimental design is the orthogonal table. According to the requirements of the number of factors, the level of factors, and whether interaction among them occurs, the participants looked up the corresponding orthogonal table, and selected representative points from a comprehensive test based on the orthogonality of the table. By doing so, it became possible to achieve results equivalent to those of a large number of full tests using the minimum number of tests, Therefore, the orthogonal table design is an efficient and economical multi-factor test design method. The distribution of orthogonal test points is shown in Figure 4.

According to the research interval shown in Table 4, the three factor levels were determined as shown in Table 5. The SPSS experimental design software was used to construct the orthogonal experiment design scheme. In the analysis of variance of the orthogonal experiment, leaving an appropriate blank column improves the accuracy of the results. Thus, L_64_3^14^ was used to arrange the orthogonal experiment table and the 14th column was left out. The test scheme and results are shown in Table 6.

### 4.1. Range Analysis

**Figure 5:**
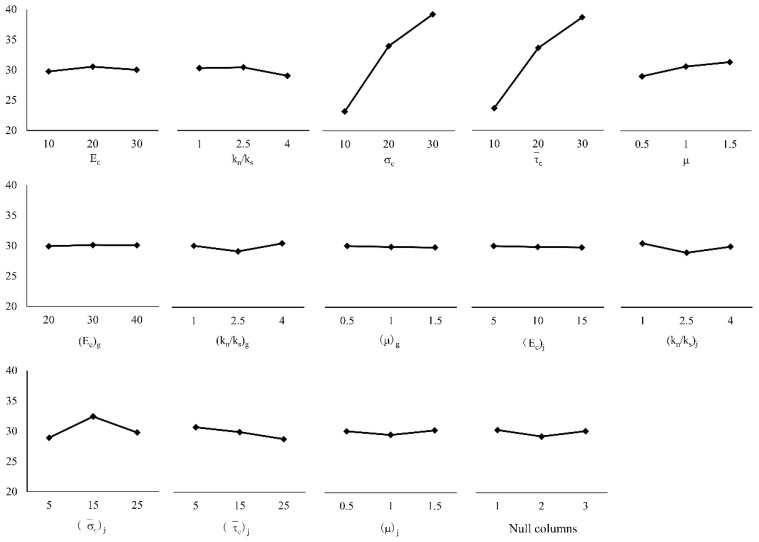
Mean value of peak intensity.

**Figure 6:**
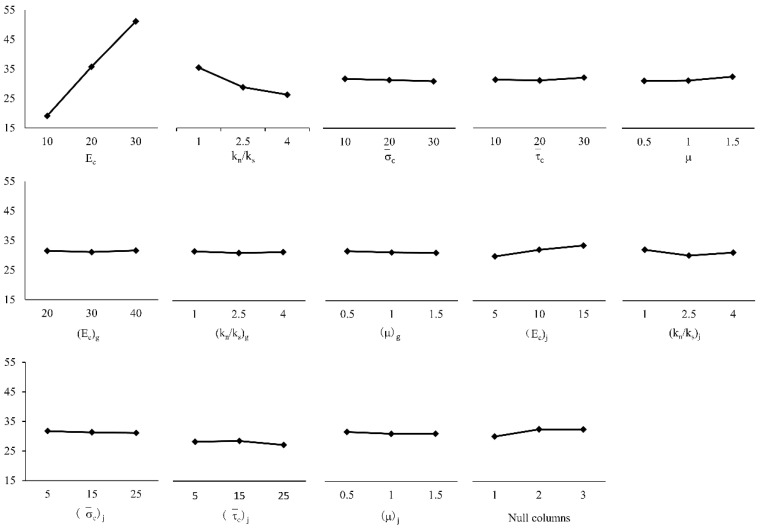
Mean modulus of elasticity.

**Figure 7:**
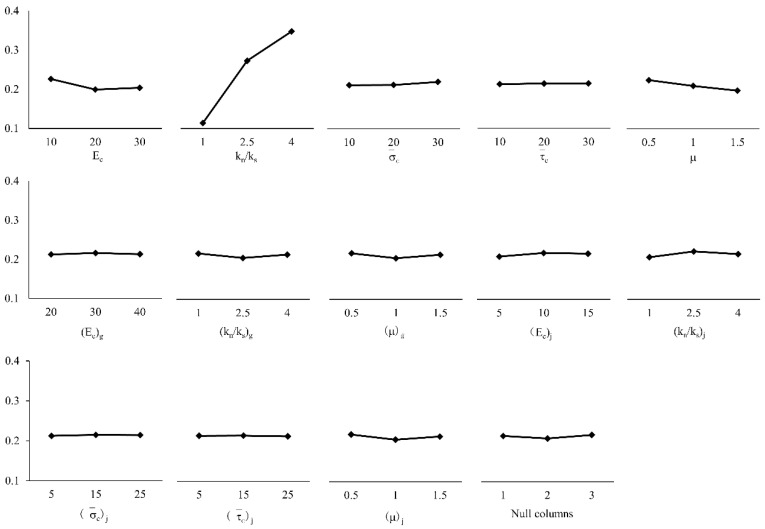
Mean value of Poisson’s ratio.

**Figure 8:**
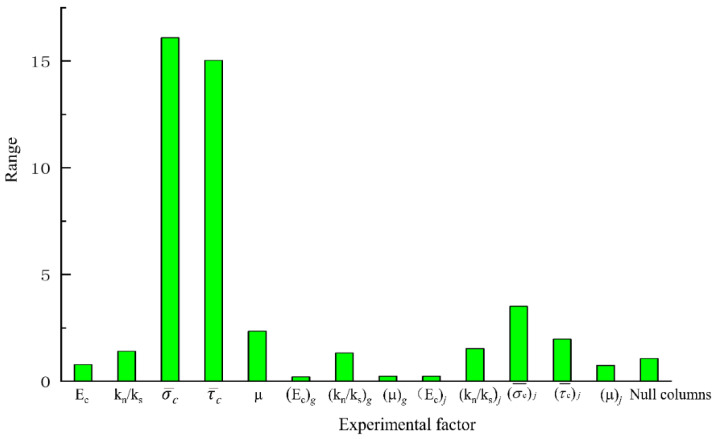
Results of analysis of range of peak intensity.

**Figure 9:**
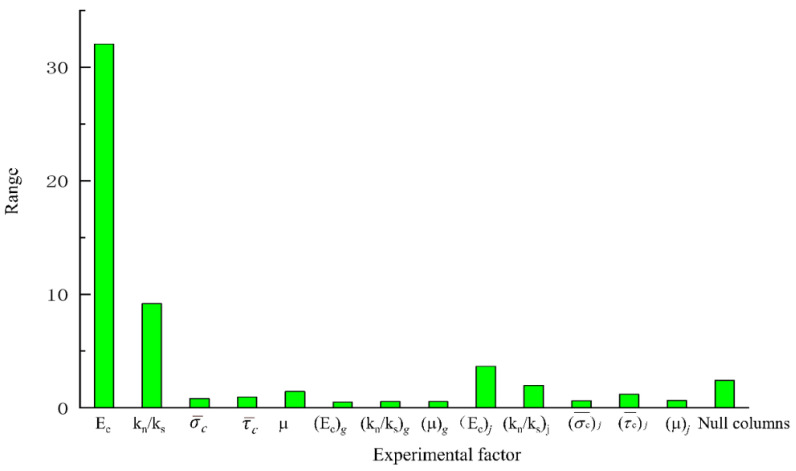
Results of analysis of range of elastic modulus.

**Figure 10:**
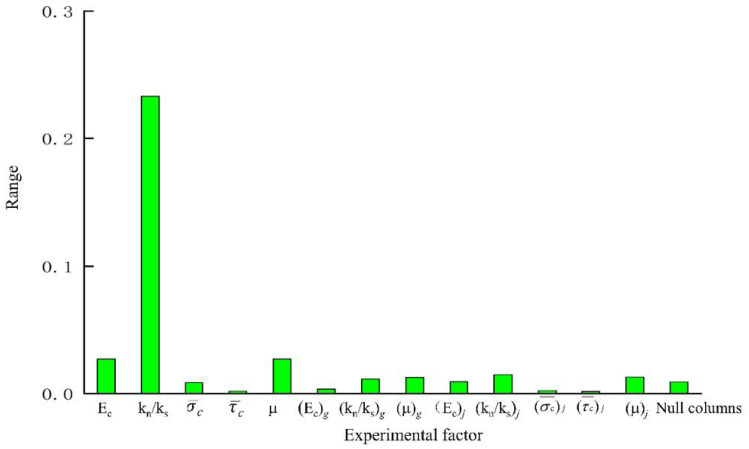
Results of analysis of range of Poisson’s ratio.

Range analysis is also called the direct analysis method. It has the advantages of simple calculation, a visual representation, and the ease of understanding of the results. It is the most commonly used method for analyzing the results of orthogonal experiments. The trends of changes in the test index average effect are shown in Figure 5, Figure 6 and Figure 7. A simple calculation can be used to obtain the influence of the factors on the test index. Range analysis was carried out using Minitab, and the results were plotted (Figure 8, Figure 9 and Figure 10).

The results show that ${\bar{\sigma }}_{c}$ and ${\bar{\tau }}_{c}$ had a significant influence on *σ_u_* because their sizes determined the difficulty of relative sliding between particles after bond failure. *E_c_* and *k_n_*/*k_s_*, which are directly related to the bond stiffness coefficient, had a significant influence on *E*, *k_n_*/*k_s_* and *μ* influenced *ν*.

### 4.2. Analysis of Variance

Range analysis of the orthogonal experimental design has the advantages of simplicity and requiring a small number of calculations, but it cannot estimate the size of the error or the importance of the influence each factor on the results. To compensate for the deficiencies of range analysis, the analysis of variance was used to analyze the results of the orthogonal experiment (Table 7).

The results of the analysis of variance were as follows:

(1) ${\bar{\sigma }}_{c}$ and ${\bar{\tau }}_{c}$ had a significant influence on *σ_u_* while the influence of other parameters was not significant.
(2) *E_c_*, *k_n_*/*k_s_*, *μ*, (*E_c_*)*_j_* and (*k_n_*/*k_s_*)*_j_* had a significant influence on *E* while the influence of other parameters was not significant.
(3) *E_c_*, *k_n_*/*k_s_*, *μ* and (*k_n_*/*k_s_*)*_j_* had a significant influence on *ν* in the interfacial transition zone of the mortar particles.

Combined with the above analysis, the relationship can be simplified as follows:

(11) $$\sigma_{u}=f_{\sigma }({\bar{\sigma }}_{c},{\bar{\tau }}_{c})$$

(12) $$E=f_{E}(E_{c},k_{n}/k_{s},\mu ,{\left(E_{c}\right)}_{j},{\left(k_{n}/k_{s}\right)}_{j})$$

(13) $$\nu =f_{\nu }(E_{c},k_{n}/k_{s},\mu ,{\left(k_{n}/k_{s}\right)}_{j})$$

The degree of fitting R^2^ of each fitting equation was between 0.7598 and 0.9777, indicating that the fitting effect was relatively good. However, due to limitations on the number and form of distribution of test points in the orthogonal experimental design, the Equation in Table 8 cannot accurately predict the macro–micro parameter relationship beyond the test points. Therefore, to increase the accuracy of the fitting Equation, another numerical test was needed.

## 5. Response Surface Design

Response surface design is a statistical method used to solve multi-variable problems by using reasonable experimental design methods, obtaining certain data through experiments, and using multiple quadratic regression equations to fit the functional relationships between the factors and the response values. and requires two to seven factors for a range of tests to identify nonlinear relationships (second order). It can be used to carry out tests with two to seven factors, and to estimate the nonlinear relationship (second order) between the test index and the factors. The fitting equation is as shown in Equation (14):

(14) $$y=\beta_{0}+\sum_{j=1}^{p}\beta_{0}x_{j}+{\sum_{i<j}\beta }_{ij}x_{i}x_{j}+\sum_{j=1}^{p}\beta_{jj}x_{j}{}^{2}+\varepsilon$$

where *β* is an undetermined parameter, *x*_k_ is the *k*_th_ independent variable, *p* is the number of factors, and *ε* is the error term.

To calculate the test factors and indicators in general, the quadratic regression equation between the levels of each factor number should be greater than or equal to three. Using a comprehensive design to estimate the above undetermined parameters leads to the problem of too much residual freedom and too high a cost. Response surface design, by testing only reasonable arrangements, avoids these problems. The Box-Behnken test is a common response surface design.

### 5.1. Central Composite Test

**Figure 11:**
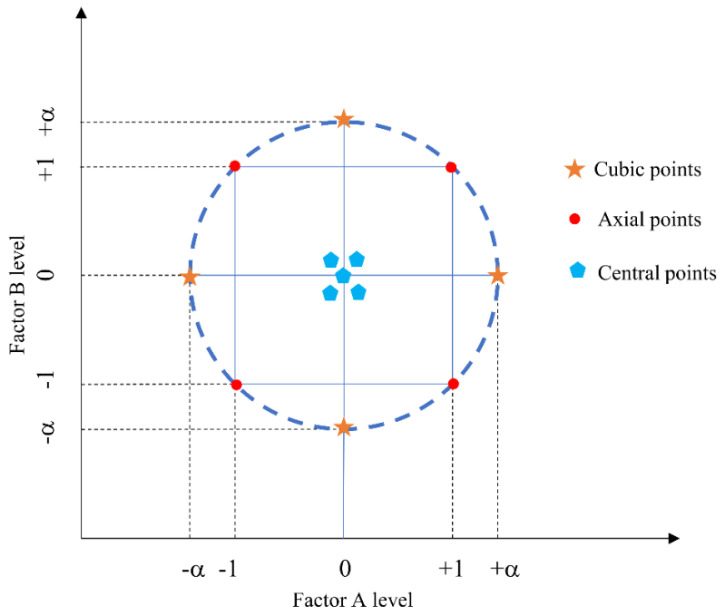
Point distribution diagram for CCD test.

As shown in Figure 11, the test point designed for the two-factor center composite test consisted of three parts: cubic points, axial points, and center points. Cubic points represent the two-level factorial design, center points represent the nonlinear test and error estimates, and axial points consider conditions of the nonlinear effect.

To enable the design to meet the requirements of rotation and sequencing, the factor level of the axial point is usually calculated according to Equation (15) [13]:

(15) $$\alpha =2^{k/4}$$

where *α* is the number of factors.

### 5.2. Box-Behnken Experimental Design

**Figure 12:**
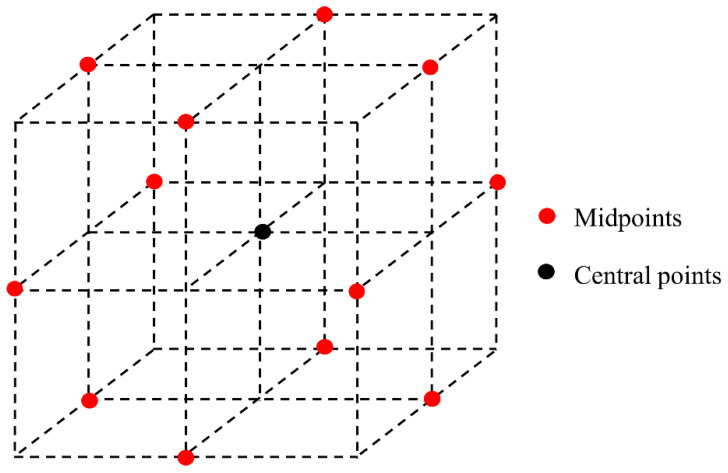
Site distribution diagram of BBD test.

Compared with the central composite test mentioned above, the Box-Behnken test has fewer tests designed with the same number of factors. There is no axial point in the test design, because of which its horizontal setting does not exceed the range of safe operation. Axial points generated by the central composite test with axial points may exceed the error threshold for safe operation area. As is shown in Figure 12, the Box-Behnken test takes each test point as the middle point of each edge of the cube and uses the center point to estimate the test error.

### 5.3. Test Plan

According to Equation (11), two factors that had a significant impact on the peak intensity corresponding to its response surface. Therefore, a two-factor CCD test table with 13 test sites (Table 9) was used for the experiment. According to Equations (12) and (13), the elastic modulus and Poisson’s ratio had significant impact factors of five and four. For the response surface corresponding to the elastic modulus and Poisson’s ratio, five and four variables were considered respectively. Considering that the axial point generated by CCD exceeded the actual value represented by the factors, a five-factor BBD test table (Table 6) with 46 test points and a four-factor BBD test table (Table 7) with 27 test points were selected for the experiment. In Table 9, point type “0” represents the center, “−1” represents the axial point, and “1” represents the cubic point. In Table 10 and Table 11, point type “0” represents the center point and type “2” represents the middle point. The other factors that had no significant influence on *σ_u_*, *E* and *ν* are not discussed. Their values were (*E_c_*)*_g_* = 30 GPa, (*k_n_*/*k_s_*)*_g_* = 2, *μ_g_* = 1, (${\bar{\sigma }}_{c}$)*_j_* = 15 MPa, (${\bar{\tau }}_{c}$)*_j_* = 15 MPa, and *μ_j_* = 1.

### 5.4. Quantitative Equation

We used the test design scheme of Minitab to design the response surface experiment (Table 9, Table 10 and Table 11), and the results were imported for response surface analysis to obtain the second-order response surface Equation of *σ_u_*, *E* and *ν*, as shown in Table 12.

The table shows that the degree of fitting of each response surface *σ_u_*, *E* and *ν* was greater than 0.988, indicating a good fitting effect and adequate reflection of information on the test site.

## 6. Calibration Program for Microscopic Parameters

The mesoscopic parameters of the PFC2D model cannot be obtained directly. Multiple numerical simulations are needed to determine them corresponding to the macroscopic physical parameters through continual selection and trial calculations to establish the relationship between them. This process is called parameter calibration. Microscopic parameter calibration is essentially a mapping problem between macroscopic and microscopic parameters. As long as a qualitative or quantitative relationship between them can be identified, the macroscopic parameters of the model can be calculated from its microscopic parameters, and the latter can be matched based on the former. We use an optimization method to calibrate the mesoscopic parameters. Based on the macro - meso parameters obtained from the previous section, the calibration problem is transformed into an optimization problem on the basis of the quantitative relationship between the mesoscopic parameters. The characteristics of the objective function and constraints on it are used to choose the appropriate optimization algorithm.

### 6.1. Optimization Model and Algorithm

The purpose of mesoscopic parameter calibration is to find a set of appropriate mesoscopic parameters for the particle discrete element model for concrete. We assumed that the macroscopic parameters of the granular discrete element concrete model derived from an empirical Equation or a quantitative relation are y_i_^*^ (*i* = 1, 2, 3……), and those of concrete measured through laboratory tests are *y_i_^lab^* (*I* = 1, 2, 3…,*s*). Then, the goal of mesoscopic parameter calibration can be converted into finding a set of mesoscopic parameters *x*_1_, *x*_2_, *x*_3_, …, *x_k_* (*k* is the total number of microscopic parameters involved) such that the difference between y_i_^*^ and *y_i_^lab^* is as small as possible. Considering the different orders of magnitude of various macroscopic parameters, the dimensionless number for relative deviation |y_i_^*^ − *y_i_^lab^*|/*y_i_^lab^* is used to describe the difference between y_i_^*^ and *y_i_^lab^*. The ultimate goal is to find a set of microscopic parameters *x*_1_, *x*_2_, *x*_3_, …, *x_k_* that minimizes the relative deviation of all macroscopic parameters.

Finally, we set the range of values of each mesoscopic parameter to [*a_j_*,*b_j_*](*j* = 1, 2, 3,…,k).

The general form of the calibration model for mesoscopic parameters is then given by:

Objective function:

(16) $$\min f(x)=\sum_{i=0}^{s}\left|\frac{y_{i}{}^{*}-y_{i}{}^{lab}}{y_{i}{}^{lab}}\right|$$

Constraint: $a_{j}\leq x_{j}\leq b_{j\;\;\;\;\;\;\;\;}(1\leq j\leq k)$

According to the above numerical test and analysis, the fitting Equation for the macroscopic parameters *σ_u_*, *E* and *ν* in the specified domain were obtained (Table 12). The microscopic parameters used as variables included $E_{c}$, $k_{n}/k_{s}$, $\bar{\sigma_{c}}$, $\bar{\tau_{c}}$, μ, ${\left(E_{c}\right)}_{j}$ and ${\left(k_{n}/k_{s}\right)}_{j}$. We set *σ_u_*, *E* and *ν* as macroscopic parameters for y1, y2, and y3, and used the parameters $E_{c}$, $k_{n}/k_{s}$, $\bar{\sigma_{c}}$, $\bar{\tau_{c}}$, μ, ${\left(E_{c}\right)}_{j}$ and ${\left(k_{n}/k_{s}\right)}_{j}$ for x1, x2, x3, x4, x5, x6, and x7 to obtain the optimal model as follows:

Objective function:

(17) $$minf(x)=\left|\frac{\sigma_{u}-\sigma_{u}{}^{*}}{\sigma_{u}{}^{*}}\right|+\left|\frac{E-E^{*}}{E^{*}}\right|+\left|\frac{\mu -\mu^{*}}{\mu^{*}}\right|$$

Constraint:

(18) $$\left\{\begin{array}{l} 10\leq x_{1}\leq 30\\ 1\leq x_{2}\leq 4\\ 10\leq x_{3}\leq 30\\ 10\leq x_{4}\leq 30\\ 0.5\leq x_{5}\leq 1.5\\ 5\leq x_{6}\leq 15\\ 1\leq x_{7}\leq 4 \end{array}\right.$$

### 6.2. Implementation of Microscopic Parameter Calibration Program

MATLAB is among the most widely used scientific computing software, and is powerful and easy to use. It provides the user with the freedom of an interactive programming interface as well as strong functional encapsulation in the form of a toolbox for users. Our work here required the MATLAB’s built-in genetic algorithm toolbox.

The algorithm used was the default one for the fitness function in the genetic algorithm toolbox. The lower the smallest value of the individual fitness value is, the close the given individual is to the optimum solution. The fitness function of the problem of microscopic parameter calibration has the same form as the objective function in Equation (17), which can be expressed as Equation (19):

Fitness function:

(19) $$Fit(x)=\left|\frac{\sigma_{u}{}^{lab}-\sigma_{u}{}^{*}}{\sigma_{u}{}^{*}}\right|+\left|\frac{E^{lab}-E^{*}}{E^{*}}\right|+\left|\frac{\mu^{lab}-\mu^{*}}{\mu^{*}}\right|$$

where *x* is an independent variable (*x* = [$E_{c}$, $k_{n}/k_{s}$, $\bar{\sigma_{c}}$, $\bar{\tau_{c}}$, μ, ${\left(E_{c}\right)}_{j}$, ${\left(k_{n}/k_{s}\right)}_{j}$]), $Fit\left(x\right)$ is the fitness value, $\sigma_{u}{}^{lab}$ is a constant obtained by the peak intensity of an indoor uniaxial compression test of rocks, $E^{lab}$ is a constant representing the elastic modulus measured using the same test, $\nu^{lab}$ is a constant representing Poisson’s ratio, and $\sigma_{u}{}^{*}$, $E^{*}$ and $\nu^{*}$ are dependent variables calculated by the quantitative Equation in Table 12.

The genetic algorithm toolbox allows the user to give equality, inequality, and boundary constraints. According to the optimization model given in Equation (17), only the boundary constraints were set to limit the upper and lower limits of each mesoscopic parameter, as shown in Equation (20):

(20) $$\mathrm{Constraint}:\;\left\{\begin{array}{l} 10\leq E_{c}\leq 30\\ 1\leq k_{n}/k_{s}\leq 4\\ 10\leq \bar{\sigma_{c}}\leq 30\\ 10\leq \bar{\tau_{c}}\leq 30\\ 0.5\leq \mu \leq 1.5\\ 5\leq {\left(E_{c}\right)}_{j}\leq 15\\ 1\leq {\left(k_{n}/k_{s}\right)}_{j}\leq 4 \end{array}\right.$$

The evolutionary process in the genetic algorithm is one of iterative optimization completed by the evolution of the population from one generation to the next. Each generation of population consists of several individuals, and the “excellence” of individuals or the fitness of the environment is evaluated by the fitness function. “Excellent” individuals are chosen for the crossover and mutation operator to form a new generation of the population, whereas suboptimal individuals are “erased.” Each generation forms a new group in this way. The optimal solution is obtained when the process of evolution of the individuals is completed.

**Figure 13:**
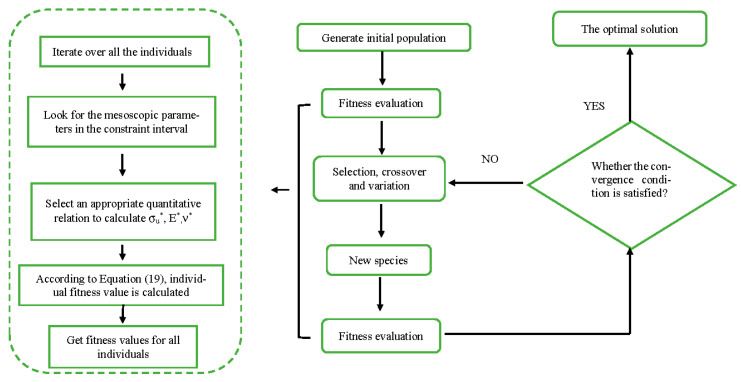
The flow of the algorithm to calibrate the microscopic parameters.

The process of solving the optimization model shown in Equation (17) by using the genetic algorithm is described as follows (Figure 13):

(1) The initial population {*x*}^0^ is generated, and *N* individuals are randomly generated (300 in this paper).
(2) Fitness evaluation is performed for each individual in the population:
  ① Assume that k = 1.
  ② Judge the interval of each microscopic parameter in the $k^{th}$ individual ${\left\{x_{k}\right\}}^{0}$.
  ③ Select the quantitative relation between the calculated $\sigma_{u}{}^{*}$, $E^{*}$ and $\nu^{*}$.
  ④ According to Equation (19), calculate the fitness value of individual ${\left\{x_{k}\right\}}^{0}$.
  ⑤ k = k+1.
  ⑥ If k≤N, continue the calculation; if k>N, exit the calculation as it is complete.
(3) Select excellent individuals and deposit them in the mating pool.
(4) Use the crossover and mutation operators to form the population ${\left\{x\right\}}^{i}$ for the next generation in the mating pool.
(5) Repeat the steps in (2) to evaluate the fitness of individuals in the new population;
(6) If the end condition is met, stop and obtain the optimal solution; otherwise, go to step (3) to continue the calculation.

MATLAB’s built-in graphical user interface (GUI) module can help users write interactive programs. Based on the MATLAB environment, the author wrote the mesoscopic parameter calibration program and designed its interface based on the GUI module. To enable the program to run independently of MATLAB’s operational environment, the MATLAB complier module was used to encapsulate the above program, and its icon and interface were designed and added. Finally, a calibration program for the microscopic parameters that could run independently was obtained.

### 6.3. Verifying Effect of Calibration Program

Table 9 and Table 10 are used to assess the range of calculation of the microscopic parameter calibration program, which is shown in Table 13.

Five groups of macroscopic parameters were selected for parameter calibration, and the corresponding optimal solution was obtained from the above program (Table 14).

When the obtained microscopic parameters were input into the DEM, the random characteristics of particle structure and aggregate distribution led to differences in the results of the simulation even if the microscopic parameters of the model remained consistent. The results were thus calculated five times to ensure the accuracy of the verification test.

Table 15 shows that the coefficients of variation of the various macroscopic parameters measured by the uniaxial compression numerical test of the concrete model were between 2.0928% and 6.3849%, indicating that the calculated mechanical parameter calculations were stable. The relative error between the measured macroscopic parameters and the target macroscopic parameters is between 0.0093%~6.4246%, which has achieved a very good calibration effect. This verified the effect of calibration and precision of the program to calibrate the microscopic parameters.

## 7. Conclusions

This paper proposed and tested a discrete element model of concrete using the particle discrete element software PFC2D. Based on the ideas of an orthogonal experimental design and response surface design, a set of mapping relationships between the macro-micro parameters of concrete were obtained. The MATLAB environment was used to write a procedure to calibrate the mesoscopic parameters. The work here can serve as a reference for future research. The main conclusions of this study are as follows:

(1) An orthogonal test of the analysis of variance showed that $\bar{\sigma_{c}}$ and $\bar{\tau_{c}}$ had a significant influence on $\sigma_{u}$, while the influence of the other parameters was not significant. $E_{c}$, $k_{n}/k_{s}$, μ $\;{\left(E_{c}\right)}_{j}$ and ${\left(k_{n}/k_{s}\right)}_{j}$ had a significant influence on E, and $E_{c}$, $k_{n}/k_{s}$, μ and ${\left(k_{n}/k_{s}\right)}_{j}$ had a significant influence on ν in the interfacial transition zone of mortar particles, while the influence of the other parameters was not significant.
(2) The response surface design method was adopted to obtain a set of quantitative relations of the macro - meso parameters of concrete within a certain range that accurately described the nonlinear relationship between them. In addition, by increasing their range of values, the mesoscopic parameters could be calibrated more precisely and, thus, more accurately inverted.
(3) The selection of an appropriate contact model of concrete, test design method, mathematical model, and optimization algorithm is key to achieving good parameter calibration.

The research results of this paper aim to provide a parameter inversion method. Due to reasons of time, test equipment and so on, no real concrete block testing was carried out in the laboratory, and only the numerical simulation part was done. In this paper, for the convenience of calculation, some parameters of the model are selected in advance, and only 13 meso-parameters within a certain range were selected as test factors. If possible, we will continue to study the relationship between other meso-parameters and macro-parameters in the future. In addition, this paper only selects concrete in the case of aggregate gradation for research, which only provides a method of mutual inversion between macro and micro parameters of concrete. If other aggregate gradations are used, this model is also applicable according to the research ideas in this paper. If we simulate materials such as reinforced concrete, more problems need to be considered. Although the modeling will be more complex, the method in this paper is also applicable. In the future, we plan to conduct further research to solve more practical problems.

## Figures and Tables

**Table 1:** Model parameters.

*L* × *W*/ mm^2^	n	$\bar{R}$/ mm	*R*_max_/*R*_min_	$\rho_{ball}\;\mathrm{kg}/m^{3}$	$\bar{\lambda }$	$\nu^{w}/\;m/s$
200 × 100	0.12	0.5	2.5	2000	1	0.02

**Table 2:** Particle size distribution and content of aggregate in concrete.

Particle Size Range/mm	5~7	7~9	9~11	11~13	13~15
Number of particles	59	28	15	8	4

**Table 3:** Values of microparameters of model.

Variety	Variable	Value
Mortar-mortar interface	*E_c_*/GPa	16
	*k_n_*/*k_s_*	1.45
	${\bar{\sigma }}_{c}$ /MPa	10
	$\bar{\tau_{c}}/\mathrm{MPa}$	30
	*μ*	0.5
Aggregate-aggregate interface	${\left(E_{c}\right)}_{g}/\mathrm{GPa}$	20
	${\left(k_{n}/k_{s}\right)}_{g}$	2
	$\mu_{g}$	1
Aggregate-mortar interface	${\left(E_{c}\right)}_{j}/\mathrm{GPa}$	8
	${\left(k_{n}/k_{s}\right)}_{j}$	1.45
	${\left(\bar{\sigma_{c}}\right)}_{j}/\mathrm{MPa}$	5
	${\left(\bar{\tau_{c}}\right)}_{j}/\mathrm{MPa}$	15
	$\mu_{j}$	0.5

**Table 4:** Research interval of microparameters.

Variety	Variable	Value Range
Mortar-mortar interface	$E_{c}/\mathrm{GPa}$	10~30
	$k_{n}/k_{s}$	1~4
	$\bar{\sigma_{c}}/\mathrm{MPa}$	10~30
	$\bar{\tau_{c}}/\mathrm{MPa}$	10~30
	μ	0.5~1.5
Aggregate-aggregate interface	${\left(E_{c}\right)}_{g}/\mathrm{GPa}$	20~40
	${\left(k_{n}/k_{s}\right)}_{g}$	1~4
	$\mu_{g}$	0.5~1.5
Aggregate-mortar interface	${\left(E_{c}\right)}_{j}/\mathrm{GPa}$	5~15
	${\left(k_{n}/k_{s}\right)}_{j}$	1~4
	${\left(\bar{\sigma_{c}}\right)}_{j}/\mathrm{MPa}$	5~25
	${\left(\bar{\tau_{c}}\right)}_{j}/\mathrm{MPa}$	5~25
	$\mu_{j}$	0.5~1.5

**Table 5:** Horizontal table of design factors of orthogonal experiment.

Factor Levels	1	2	3
$E_{c}/\mathrm{GPa}$	10	20	30
$k_{n}/k_{s}$	1	2.5	4
$\bar{\sigma_{c}}/\mathrm{MPa}$	10	20	30
$\bar{\tau_{c}}/\mathrm{MPa}$	10	20	30
μ	0.1	1	1.5
${\left(E_{c}\right)}_{g}/\mathrm{GPa}$	20	30	40
${\left(k_{n}/k_{s}\right)}_{g}$	1	2.5	4
$\mu_{g}$	0.5	1	1.5
${\left(E_{c}\right)}_{j}/\mathrm{GPa}$	5	10	15
${\left(k_{n}/k_{s}\right)}_{j}$	1	2.5	4
${\left(\bar{\sigma_{c}}\right)}_{j}/\mathrm{MPa}$	5	15	25
${\left(\bar{\tau_{c}}\right)}_{j}/\mathrm{MPa}$	5	15	25
$\mu_{j}$	0.5	1	1.5

**Table 6:** Orthogonal test scheme and results.

Serial Number	$\begin{array}{l} E_{c}/\\ GPa \end{array}$	$\begin{array}{l} k_{n}/k_{s} \end{array}$	$\begin{array}{l} \bar{\sigma_{c}}/\\ MPa \end{array}$	$\begin{array}{l} \bar{\tau_{c}}/\\ MPa \end{array}$	$\begin{array}{l} \mu \end{array}$	$\begin{array}{l} {\left(E_{c}\right)}_{g}/\\ GPa \end{array}$	$\begin{array}{l} {\left(k_{n}/k_{s}\right)}_{g} \end{array}$	$\mu_{g}$	$\begin{array}{l} {\left(E_{c}\right)}_{j}/\\ GPa \end{array}$	$\begin{array}{l} {\left(k_{n}/k_{s}\right)}_{j} \end{array}$	$\begin{array}{l} {\left(\bar{\sigma_{c}}\right)}_{j}/\\ MPa \end{array}$	$\begin{array}{l} {\left(\bar{\tau_{c}}\right)}_{j}/\\ M\mathrm{pa} \end{array}$	$\begin{array}{l} \mu_{j} \end{array}$	Null Columns	$\begin{array}{l} \sigma_{u}/\\ MPa \end{array}$	$\begin{array}{l} E/\\ GPa \end{array}$	ν
1	20	1	10	10	1.5	20	2.5	0.5	5	2.5	15	25	0.5	3	21.74	38.22	0.100
2	10	1	30	30	0.5	20	4	1.5	10	2.5	15	5	0.5	1	53.96	21.29	0.158
3	10	2.5	20	20	0.5	30	1	0.5	15	2.5	5	25	1.5	2	37.56	18.17	0.316
4	20	1	30	30	0.5	20	1	0.5	5	1	15	5	1.5	2	56.86	37.29	0.112
5	30	1	20	10	1	30	1	1	5	2.5	15	5	0.5	2	24.27	53.38	0.110
6	10	2.5	30	10	1.5	20	1	1	10	1	5	25	0.5	2	30.63	18.55	0.266
7	10	1	10	20	1.5	30	1	1	5	4	5	5	1.5	1	26.86	20.69	0.104
8	10	1	10	10	1	20	1	1	15	1	15	25	1.5	1	20.81	23.42	0.116
9	20	1	20	20	1	20	1	1.5	10	1	5	25	0.5	3	42.12	42.16	0.094
10	20	2.5	20	30	0.5	40	2.5	1.5	5	1	5	15	0.5	1	44.38	31.40	0.261
11	30	4	20	20	0.5	40	2.5	1	5	1	5	25	0.5	1	37.91	40.60	0.322
12	10	4	30	30	1	40	1	1	5	4	15	5	0.5	3	56.01	15.07	0.370
13	30	1	10	30	1.5	40	4	0.5	15	1	5	5	0.5	2	27.70	64.53	0.085
14	20	1	10	10	0.5	20	2.5	1	15	4	5	5	1	2	20.37	40.96	0.012
15	10	1	10	10	1.5	20	1	1.5	10	1	25	15	1	1	21.85	23.09	0.095
16	10	4	10	20	1.5	20	2.5	0.5	5	4	15	15	0.5	2	22.94	15.33	0.342
17	10	4	20	30	0.5	30	1	0.5	10	2.5	5	15	1	2	38.73	15.99	0.383
18	30	2.5	30	30	1.5	30	2.5	0.5	15	1	15	5	1	1	62.41	51.82	0.256
19	10	4	10	10	0.5	40	4	1	5	1	25	15	1.5	2	20.95	15.43	0.343
20	30	1	30	20	0.5	20	1	0.5	5	1	25	5	1	2	40.65	53.43	0.112
21	10	1	30	10	0.5	30	2.5	1.5	5	1	5	25	0.5	1	24.53	20.46	0.122
22	30	1	30	10	0.5	40	1	0.5	10	4	5	15	1	3	24.44	56.35	0.139
23	10	2.5	20	10	0.5	20	1	0.5	5	1	15	5	1	3	25.96	17.01	0.275
24	10	1	30	20	0.5	20	4	1	15	2.5	25	5	0.5	1	40.87	22.07	0.160
25	10	1	20	10	1	40	2.5	0.5	15	1	25	5	1	1	24.58	23.40	0.119
26	20	1	10	20	1	40	4	0.5	10	1	5	5	0.5	2	26.64	43.96	0.105
27	20	1	20	10	1.5	30	1	1.5	5	2.5	25	5	0.5	2	25.79	38.25	0.095
28	20	1	30	10	0.5	40	1	0.5	15	4	5	25	1.5	3	24.93	41.63	0.147
29	10	1	20	30	1	20	4	0.5	5	4	5	15	1.5	1	48.30	20.38	0.122
30	10	4	10	10	1.5	30	4	0.5	10	1	5	5	0.5	3	20.23	16.68	0.347
31	20	2.5	30	10	1.5	20	4	0.5	5	2.5	5	15	1.5	1	30.86	31.66	0.247
32	10	4	20	10	0.5	20	1	0.5	5	1	25	5	1.5	3	26.04	15.39	0.347
33	30	2.5	10	20	0.5	20	1	0.5	5	1	5	5	0.5	1	22.99	44.78	0.254
34	20	4	20	10	0.5	20	4	1.5	15	4	15	5	0.5	1	25.98	31.11	0.366
35	10	1	10	10	0.5	20	1	0.5	5	1	5	5	0.5	1	20.15	20.47	0.124
36	10	2.5	30	20	1.5	40	1	1.5	5	4	25	5	0.5	3	49.30	16.93	0.276
37	10	1	10	10	0.5	20	1	0.5	5	1	5	5	0.5	1	20.15	20.47	0.124
38	30	4	10	30	1.5	20	1	1.5	15	1	25	25	1.5	1	24.16	47.83	0.325
39	10	4	10	20	0.5	20	2.5	1.5	15	2.5	5	5	1	3	20.35	16.49	0.381
40	20	1	10	30	0.5	30	4	1	5	1	25	25	1	3	30.09	37.85	0.110
41	10	1	20	10	1.5	40	2.5	0.5	10	1	15	5	1.5	1	25.77	23.06	0.096
42	30	2.5	10	10	1	40	1	1.5	5	2.5	5	5	1.5	1	21.03	44.48	0.251
43	30	1	20	30	1.5	20	1	1	15	1	5	15	0.5	3	50.86	64.18	0.085
44	10	2.5	10	30	0.5	20	2.5	1	10	2.5	5	5	1.5	3	22.85	17.68	0.300
45	10	2.5	10	10	0.5	40	4	1.5	5	1	15	25	1	2	20.68	16.99	0.273
46	30	4	10	10	0.5	30	1	0.5	10	4	15	25	0.5	1	21.17	45.68	0.352
47	20	2.5	10	10	0.5	30	1	0.5	15	4	25	15	0.5	1	21.42	34.27	0.296
48	30	1	10	20	0.5	30	4	1.5	5	1	15	15	1.5	3	27.53	53.99	0.106
49	10	2.5	10	30	1	20	2.5	0.5	5	4	25	25	0.5	2	25.38	16.82	0.272
50	10	1	10	20	0.5	40	1	0.5	15	2.5	15	15	0.5	1	26.90	22.22	0.153
51	20	4	10	30	0.5	20	1	0.5	5	1	5	5	0.5	1	21.06	28.43	0.330
52	30	4	30	10	1	20	4	0.5	5	2.5	5	25	1	1	29.19	40.27	0.324
53	10	1	10	30	1	30	1	1.5	5	4	5	5	1	1	27.12	20.43	0.116
54	10	1	30	10	0.5	30	2.5	1	5	1	5	15	0.5	1	24.54	20.46	0.123
55	10	4	30	10	1	20	1	1.5	15	1	5	15	0.5	2	29.22	16.86	0.366
56	20	4	10	10	1.5	40	1	1	5	2.5	5	5	1	1	20.69	28.55	0.323
57	30	2.5	20	10	0.5	20	4	1	10	4	25	5	0.5	1	26.11	46.86	0.285
58	10	1	20	20	1.5	20	4	0.5	5	4	5	25	1	1	39.86	20.56	0.114
59	20	2.5	10	20	1	20	1	1	10	1	15	15	1	1	25.58	34.85	0.253
60	30	1	10	10	0.5	20	2.5	1.5	10	4	5	5	1.5	2	19.98	56.56	0.139
61	20	4	30	20	1	30	2.5	0.5	10	1	25	5	1.5	1	49.94	30.98	0.337
62	30	1	10	10	1	20	2.5	0.5	5	2.5	25	15	0.5	3	20.01	53.50	0.112
63	10	2.5	10	10	1	30	4	0.5	15	1	5	5	0.5	3	19.90	18.89	0.284
64	10	1	10	30	0.5	40	1	0.5	10	2.5	25	25	0.5	1	29.37	21.46	0.147

**Table 7:** Results of analysis of variance.

Factors	$\sigma_{u}/MPa$		E/GPa		ν/−	
	F	Significance	F	Significance	F	Significance
$E_{c}/\mathrm{GPa}$	0.17	-	3003.42	Highly significant	12.84	Highly significant
$k_{n}/k_{s}$	0.22	-	248.39	Highly significant	1117.1	Highly significant
$\bar{\sigma_{c}}/\mathrm{MPa}$	42.37	Highly significant	0.64	-	2.24	-
$\bar{\tau_{c}}/\mathrm{MPa}$	36.8	Highly significant	3.16	-	0.54	-
μ	1.15	-	8.9	Highly significant	10.48	Highly significant
${\left(E_{c}\right)}_{g}/\mathrm{GPa}$	0.05	-	0.69	-	0.75	-
${\left(k_{n}/k_{s}\right)}_{g}$	0.19	-	0.2	-	1.44	-
$\mu_{g}$	0	-	0.12	-	1.81	-
${\left(E_{c}\right)}_{j}/\mathrm{GPa}$	0	-	49.03	Highly significant	3.24	-
${\left(k_{n}/k_{s}\right)}_{j}$	0.21	-	7.28	Highly significant	5.87	Highly significant
${\left(\bar{\sigma_{c}}\right)}_{j}/\mathrm{MPa}$	2.05	-	0.2	-	0.67	-
${\left(\bar{\tau_{c}}\right)}_{j}/\mathrm{MPa}$	0.38	-	3.08	-	0.18	-
$\mu_{j}$	0.06	-	0.31	-	1.79	-

**Table 8:** Regression analysis of orthogonal experimental design.

Fitting Equation	$R^{2}\;$	Domain of Definition						
		$E_{c}/GPa$	$k_{n}/k_{s}$	$\bar{\sigma_{c}}/Mpa$	$\bar{\tau_{c}}/M\mathrm{pa}$	μ	${\left(E_{c}\right)}_{j}/GPa$	${\left(k_{n}/k_{s}\right)}_{j}$
$\sigma_{u}=-12.58+0.934\bar{\sigma_{c}}+0.7797\bar{\tau_{c}}$	0.7598	-	-	(10,30)	(10,30)	-	-	-
$E=5.15+1.6058\;E_{c}-3.015\;k_{n}/k_{s}+$ $1.691\mu +0.4063{\left(E_{c}\right)}_{j}-0.282{\left(k_{n}/k_{s}\right)}_{j}$	0.9777	(10,30)	(1,4)	-	-	(0.5,1.5)	( 5,15)	(1,4)
$\nu =-0.0725-0.001096E_{c}+0.08025\;k_{n}/k_{s}$ $-0.02369\mu +0.00424{\left(k_{n}/k_{s}\right)}_{j}$	0.9382	(10,30)	(1,4)	-	-	(0.5,1.5 )	-	(1,4)

**Table 9:** Peak intensity of the CCD test table.

Point Type	$\bar{\sigma_{c}}/Mpa$	$\bar{\tau_{c}}/M\mathrm{pa}$	$\sigma_{u}/MPa$
0	20	20	42.23
0	20	20	39.53
−1	34.142	20	52.2
1	30	10	30.24
1	30	30	62.09
−1	20	34.142	50.85
1	10	10	22.41
0	20	20	41.73
0	20	20	40.04
1	10	30	27.85
−1	5.858	20	17
−1	20	5.858	18.83
0	20	20	43.17

**Table 10:** BBD test table of the elastic modulus.

Point Type	$\begin{array}{l} E_{c}/\\ GPa \end{array}$	$\begin{array}{l} k_{n}/k_{s} \end{array}$	μ	$\begin{array}{l} {\left(E_{c}\right)}_{j}/\\ GPa \end{array}$	$\begin{array}{l} {\left(k_{n}/k_{s}\right)}_{j} \end{array}$	$\begin{array}{l} E/GPa \end{array}$
2	10	2.5	1	15	2.5	18.716
2	20	2.5	0.5	10	4	32.299
2	20	1	1	10	1	42.465
0	20	2.5	1	10	2.5	34.058
2	20	2.5	1.5	5	2.5	31.608
2	20	1	1	10	4	40.867
2	20	4	1	5	2.5	28.351
2	30	2.5	1	10	1	50.275
2	20	1	1	15	2.5	43.257
2	30	4	1	10	2.5	44.084
2	20	4	1	10	1	31.469
0	20	2.5	1	10	2.5	36.364
0	20	2.5	1	10	2.5	34.442
2	20	4	1.5	10	2.5	30.973
2	10	4	1	10	2.5	16.384
2	20	1	0.5	10	2.5	40.007
2	20	2.5	0.5	10	1	33.951
2	10	2.5	1	5	2.5	17.028
2	30	2.5	1	15	2.5	51.058
0	20	2.5	1	10	2.5	33.646
2	20	4	1	10	4	30.449
2	20	2.5	1.5	10	1	35.234
2	20	4	1	15	2.5	32.006
2	20	2.5	1	15	4	35.126
2	10	2.5	1	10	1	18.448
2	30	2.5	1	5	2.5	44.425
2	30	2.5	0.5	10	2.5	47.56
2	10	2.5	1	10	4	18.081
2	10	1	1	10	2.5	22.25
2	20	2.5	1	15	1	36.099
2	20	2.5	1.5	15	2.5	35.794
0	20	2.5	1	10	2.5	33.962
2	30	2.5	1.5	10	2.5	49.165
2	20	2.5	1	5	1	32.324
2	20	2.5	1.5	10	4	34.048
2	30	2.5	1	10	4	48.192
2	20	2.5	1	5	4	30.878
2	10	2.5	0.5	10	2.5	17.708
2	20	1	1	5	2.5	37.769
2	20	2.5	0.5	5	2.5	30.533
2	20	2.5	0.5	15	2.5	34.623
2	20	4	0.5	10	2.5	30.159
2	20	1	1.5	10	2.5	41.986
2	30	1	1	10	2.5	58.904
0	20	2.5	1	10	2.5	34.163
2	10	2.5	1.5	10	2.5	18.331

**Table 11:** BBD test table for Poisson’s ratio.

Point Type	$\begin{array}{l} E_{c}/GPa \end{array}$	$\begin{array}{l} k_{n}/k_{s} \end{array}$	$\begin{array}{l} \mu \end{array}$	$\begin{array}{l} {\left(k_{n}/k_{s}\right)}_{j} \end{array}$	$\begin{array}{l} \nu \end{array}$
2	30	2.5	1.5	2.5	0.248
2	30	2.5	1	4	0.263
2	20	1	0.5	2.5	0.131
0	20	2.5	1	2.5	0.26
2	10	2.5	1	1	0.264
2	30	2.5	0.5	2.5	0.273
2	10	2.5	0.5	2.5	0.291
2	10	2.5	1.5	2.5	0.265
2	30	1	1	2.5	0.103
2	20	4	1	1	0.324
2	20	1	1	1	0.085
0	20	2.5	1	2.5	0.256
2	20	1	1	4	0.113
0	20	2.5	1	2.5	0.213
2	20	4	0.5	2.5	0.347
2	20	4	1.5	2.5	0.33
2	20	4	1	4	0.341
2	30	2.5	1	1	0.238
2	10	4	1	2.5	0.347
2	30	4	1	2.5	0.327
2	10	1	1	2.5	0.131
2	20	2.5	0.5	1	0.267
2	20	1	1.5	2.5	0.092
2	10	2.5	1	4	0.277
2	20	2.5	1.5	4	0.261
2	20	2.5	0.5	4	0.285
2	20	2.5	1.5	1	0.241

**Table 12:** Fitting Equation of *σ_u_*, *E* and *ν*.

Response Surface Equation	$R^{2}$	Domain of Definition						
		$E_{c}/$ GPa	$k_{n}/k_{s}$	$\bar{\sigma_{c}}/Mpa$	$\bar{\tau_{c}}/M\mathrm{pa}$	μ	${\left(E_{c}\right)}_{j}/$ GPa	${\left(k_{n}/k_{s}\right)}_{j}$
$\sigma_{u}=-0.48+1.083\bar{\sigma_{c}}+0.919\bar{\tau_{c}}-$ $0.03138{\bar{\sigma_{c}}}^{2}-0.03018{\bar{\tau_{c}}}^{2}+0.06602\bar{\sigma_{c}}\times \bar{\tau_{c}}$	0.9881	-	-	(10,30)	(10,30)	-	-	-
$E=0.30+1.998E_{c}-3.647k_{n}/k_{s}+4.71\mu$ $+0.715{\left(E_{c}\right)}_{j}-0.396{\left(k_{n}/k_{s}\right)}_{j}-0.00771E_{c}{}^{2}$ $+0.7972k_{n}/k_{s}{}^{2}-1.959\mu^{2}-0.03420{\left(E_{c}\right)}_{j}{}^{2}$ $+0.0129{\left(k_{n}/k_{s}\right)}_{j}{}^{2}-0.1492E_{c}\times \;k_{n}/k_{s}$ $+0.0491E_{c}\times \mu +0.02473E_{c}\times {\left(E_{c}\right)}_{j}$ $-0.0286\;E_{c}\times {\left(k_{n}/k_{s}\right)}_{j}-0.388\;k_{n}/k_{s}\times \mu$ $-0.0611\;k_{n}/k_{s}\times {\left(E_{c}\right)}_{j}+0.064\;k_{n}/k_{s}\times$ ${\left(k_{n}/k_{s}\right)}_{j}+0.010\mu \times {\left(E_{c}\right)}_{j}$ $+0.155\mu \times {\left(k_{n}/k_{s}\right)}_{j}+0.0158{\left(E_{c}\right)}_{j}\times {\left(k_{n}/k_{s}\right)}_{j}$	0.9981	(10,30)	(1,4)	-	-	(0.5,1.5)	(5,15)	(1,4)
$\begin{array}{c} \nu =0.1499-0.00726\;E_{c}+0.1378k_{n}/k_{s}\\ -0.1582\mu -0.0057{\left(k_{n}/k_{s}\right)}_{j}+0.000134\;E_{c}{}^{2}\\ -0.01383k_{n}/k_{s}{}^{2}+0.0555\mu^{2}+0.00217 \end{array}$ ${\left(k_{n}/k_{s}\right)}_{j}{}^{2}+0.000133\;E_{c}\times \;k_{n}/k_{s}+0.00005\;$ $E_{c}\times \mu +0.000200\;E_{c}\times {\left(k_{n}/k_{s}\right)}_{j}+0.00733\;$ $k_{n}/k_{s}\times \mu -0.00122\;k_{n}/k_{s}\times {\left(k_{n}/k_{s}\right)}_{j}$ $+0.00067\mu \times {\left(k_{n}/k_{s}\right)}_{j}$	0.9911	(10,30)	(1,4)	-	-	(0.5,1.5)	-	(1,4)

**Table 13:** Calculation range of the microscopic parameter calibration program.

** Mesoscopic parameters **	$E_{c}/\mathrm{GPa}$	$k_{n}/k_{s}$	$\bar{\sigma_{c}}/\mathrm{MPa}$	$\bar{\tau_{c}}/\mathrm{MPa}$	μ	${\left(E_{c}\right)}_{j}/\mathrm{GPa}$	${\left(k_{n}/k_{s}\right)}_{j}$
	(10, 30)	(1, 4)	(10, 30)	(10, 30)	(0.5, 1.5)	(5, 15)	(1, 4)
** Macro parameters **	$\sigma_{u}/\mathrm{MPa}$	E/GPa	ν				
	(17, 62.09)	(16.384, 58.904)	(0.085, 0.347)				

**Table 14:** Five groups of parameters and their optimal solutions.

Concrete Number	Input: Macro Parameters			Output: Mesoscopic Parameters						
	$\sigma_{u}/MPa$	E/GPa	ν	$E_{c}/GPa$	$k_{n}/k_{s}$	$\bar{\sigma_{c}}/MPa$	$\bar{\tau_{c}}/MPa$	μ	${\left(E_{c}\right)}_{j}/GPa$	${\left(k_{n}/k_{s}\right)}_{j}$
1	59.67	32.68	0.201	17.522	1.983	28.700	27.884	1.356	10.236	2.626
2	42.67	26.78	0.209	13.944	1.750	18.938	22.454	0.830	10.552	3.797
3	36.54	25.66	0.218	13.435	2.000	14.277	23.109	0.867	11.360	2.205
4	47.67	21.54	0.222	11.013	1.938	24.447	21.680	0.930	11.622	2.923
5	43.6	24.67	0.222	12.815	1.980	26.243	18.139	1.292	11.263	3.783

**Table 15:** Results and errors of macroscopic parameter matching.

Concrete Number	Macro Parameters	Target Value	Macroscopic Parameters of Numerical Simulation			Relative Error/%
			Average Value	Standard Deviation	Coefficient of Variation/%	
1	$\sigma_{u}/\mathrm{MPa}$	59.67	57.74	3.5224	6.1007	3.3461
	E/GPa	32.68	34.08	1.3190	3.8701	4.1136
	ν	0.201	0.21	0.0058	2.7002	6.4246
2	$\sigma_{u}/\mathrm{MPa}$	42.67	41.24	1.8370	4.4547	3.4750
	E/GPa	26.78	26.77	0.9748	3.6419	0.0523
	ν	0.209	0.22	0.0046	2.0928	4.9136
3	$\sigma_{u}/\mathrm{MPa}$	36.54	34.34	2.1924	6.3849	6.4158
	E/GPa	25.66	25.69	1.0284	4.0031	0.1168
	ν	0.218	0.23	0.0053	2.3043	5.2174
4	$\sigma_{u}/\mathrm{MPa}$	47.67	45.45	2.7454	6.0406	4.8868
	E/GPa	21.54	21.54	0.8966	4.1629	0.0093
	ν	0.222	0.24	0.0061	2.5738	6.3291
5	$\sigma_{u}/\mathrm{MPa}$	43.6	43.09	1.5989	3.7109	1.1906
	E/GPa	24.67	24.62	0.9439	3.8339	0.2031
	ν	0.222	0.23	0.0059	2.5279	4.7210

## Data Availability

The data presented in this study are available on request the corresponding author.
